# Impact of the COVID-19 pandemic on quality of life of adults with diabetes in rural Uganda: a cross-sectional survey

**DOI:** 10.1093/inthealth/ihaf112

**Published:** 2025-10-07

**Authors:** Wenceslaus Sseguya, Silver Bahendeka, Sara MacLennan, Aravinda Meera Guntupalli

**Affiliations:** Institute of Applied Health Sciences, University of Aberdeen, Health Sciences Building, Foresterhill, Aberdeen, AB25 2ZD, UK; Department of Internal Medicine, St Francis Hospital Nsambya, P.O.Box 7146, Kampala, Uganda; Department of Internal Medicine, Mother Kevin Postgraduate Medical School, Uganda Martyrs University Medical School, P.O.Box 5498, Kampala, Uganda; Department of Internal Medicine, St Francis Hospital Nsambya, P.O.Box 7146, Kampala, Uganda; Institute of Applied Health Sciences, University of Aberdeen, Health Sciences Building, Foresterhill, Aberdeen, AB25 2ZD, UK; Institute of Applied Health Sciences, University of Aberdeen, Health Sciences Building, Foresterhill, Aberdeen, AB25 2ZD, UK

**Keywords:** COVID-19 pandemic, diabetes mellitus, quality of life, rural, Uganda

## Abstract

**Background:**

The coronavirus disease 2019 (COVID-19) pandemic was associated with unprecedented healthcare, economic and social disruptions that impacted persons with diabetes mellitus (PWDM). We aimed to establish how the quality of life (QoL) of persons with diabetes in rural Uganda was impacted by the COVID-19 pandemic, using the pre-pandemic, pandemic and post-pandemic self-reported QoL scores.

**Methods:**

We surveyed 410 PWDM ≥30 y of age from three rural districts in south-western Uganda. Median QoL scores were computed and variations across the three time periods were analysed using the Friedman analysis of variance and McNemar tests as appropriate. Logistic regression was used to identify factors associated with QoL. A p-value <0.05 indicated statistical significance.

**Results:**

The overall median QoL scores were 67.2 (pre-pandemic), 62.4 (pandemic) and 68.8 (post-pandemic) (p<0.001). There was a 75% increase in the proportion of participants with unsatisfactory QoL during the pandemic (p<0.001). Having diabetes complications (p<0.001), chronic comorbidity (p=0.012), no formal education (p<0.003) and travelling for healthcare using non-motorised transport (<0.001) were all independently associated with post-pandemic unsatisfactory QoL.

**Conclusions:**

The COVID-19 pandemic caused significant deterioration in QoL among rural PWDM, raising the need for policies to prioritise the consideration of their evolving needs while designing measures for future similar widespread emergencies.

## Introduction

Measurement of health-related quality of life (QoL) in chronic disease care has increasingly become popularly applied in patient-based care evaluations and chronic care planning. The contemporary approach to the measurement of health has shifted from focusing only on traditional health measures, such as morbidity and mortality, to a broader focus that incorporates measures of the impact of disease processes and treatment on an individual’s daily functioning and well-being.^1^ QoL measurements are multidimensional and define an individual’s perception of how disease treatments, disease processes and the wider environment in which they live impact the various aspects of life in relation to their goals and expectations.^2^ Over the past 3 decades there has been a growing application of QoL assessments in various clinical and public health settings, including in persons with diabetes mellitus (PWDM).^3^ These assessments are designed to reflect how PWDM perceive and react to the health or non-health aspects and the interaction thereof in their lives.^4^ The health-related aspects, which include physical, emotional, psychological and functional perceptions, are direct outcomes of the impact of the disease or treatment processes. Non-health aspects, which include but are not limited to social, financial and spiritual perceptions, are indirect outcomes of disease demands and/or the wider environmental circumstances surrounding an individual.^4^

Diabetes is a major contributor to the chronic disease burden, especially in low-income countries, where chronic healthcare services are largely inadequate.^5,6^ For most sub-Saharan African countries, where the double disease burden and resurgence of epidemics have recurrently taken a toll on their health system capacity, diabetes care continues to receive limited prioritisation even with the growing diabetes prevalence burden.^7–9^ Rural settings suffer the most, given their existing inequalities in the distribution, availability and accessibility of healthcare, coupled with the disproportionately high poverty rates.^10,11^ The recent coronavirus disease 2019 (COVID-19) pandemic has been documented to have exacerbated the diabetes care burden by triggering a shift in healthcare system priorities and disrupting the functioning of other sectors that are indirectly linked to health.^12^ A systematic review of QoL studies among PWDM conducted in Africa before the pandemic showed health-related QoL in PWDM to be negatively associated with rural residence, comorbidities and diabetes complications.^13^ The impact of the pandemic on QoL in PWDM has been assessed by a few studies that have reported varying impacts, with a dearth of published literature within rural contexts. Whereas in some parts, like India,^14^ no significant difference was seen between pre- and post-pandemic QoL, elsewhere the pandemic was found to have negatively impacted various aspects of QoL, such as in Ethiopia,^15^ Lebanon^16^ and Singapore.^17^ These studies have attributed this negative impact to the COVID-19 pandemic measures, but also found older age and the presence of diabetes complications and comorbidities among the factors associated with negative QoL.

Uganda imposed various lockdown measures, which widely disrupted social lives, the economy and healthcare services.^18,19^ Previous studies indicate that diabetes-related healthcare services and support were considerably impacted during the pandemic.^20,21^ Hence, we assessed the QoL as perceived by PWDM retrospectively across the pre-pandemic and pandemic periods and prospectively in the post-pandemic period to gauge the variation. We therefore provide the context through which rural PWDM in a low-income country like Uganda compare post-pandemic diabetes-related QoL with the pandemic and pre-pandemic periods.

## Methods

### Study design and participants

This was a cross-sectional quantitative survey conducted between October 2023 and April 2024 among adult diabetes patients attending primary care facilities in the rural districts of Sheema, Isingiro and Ntungamo in south-western Uganda. Participants were PWDM who were residents of the respective districts, ≥30 y of age and had been diagnosed and attending diabetes care from public primary care facilities for at least 4 y prior to the COVID-19 pandemic.

### Sample size determination

We derived the minimum sample (n) required to test our hypothesis using Taro Yamane’s formula,^22^ n=N/1+N(e)^2^, where N defines the number of persons with diabetes ≥30 y of age in the population and e is the allowable error set at the 95% confidence interval (0.05). At the time of the study, 3176 diabetes patients ≥30 y of age were registered at the various government primary care facilities across the three districts. The formula yielded a minimum required sample of 355, which provided a statistical power of >99% (α=0.05) as tested using the G*Power software (version 3.1.9.7).^23^ We recruited a final sample of 410 adult diabetes patients during the study, which represented 12.9% of the total registered diabetes patients who met our inclusion criteria, with a non-response rate of 6.2%.

### Sampling and recruitment of participants

Stratified probability sampling was used to obtain a proportionate sample of adult PWDM across the three districts. Each district formed a sampling stratum that contributed a proportionate number towards the total sample of the three districts. A diabetes clinic register from each of the 20 selected health facilities formed the sampling frame, from which participants meeting the inclusion criteria were randomly selected to make the allotted proportionate sample for each primary care facility. The patient registers were accessed through the health workers in charge of diabetes services at every primary care facility, who also assisted in identifying participants meeting the inclusion criteria. Study posters were displayed in the diabetes service areas informing PWDM of the recruitment process of eligible persons. Those identified as eligible for participation were contacted through their phone details provided in the registers. Participant information leaflets and consent forms were provided to each identified prospective participant on their subsequent clinic visit. Written consent was obtained from all participants. We recruited a significantly higher number of females than males. Given our study’s low non-response rate and the lack of a sex-related difference in diabetes prevalence within these settings,^24^ the low male recruitment was due to the gender-related variations in public healthcare service utilisation, as our participants were recruited from public healthcare facilities. In Uganda, studies have shown males have considerably higher odds of utilising private healthcare compared with females.^25^

### Data collection tools and procedures

The questionnaire was interviewer administered (by WS) face-to-face and consisted of three sections: sociodemographic characteristics, lifestyle and clinical characteristics and diabetes-related quality of life scores. The participants were interviewed at their respective primary care facilities. An online version of the survey questionnaire designed with SNAP version 11 was used by the interviewer to instantly transfer the data to a secure server under the University of Aberdeen.

### Development of the diabetes-related QoL questionnaire

The QoL questionnaire used in our study was adopted from Nagpal et al.^26^ We adopted five domains comprising 25 items measured on a 5-point Likert scale. The domains are role limitation due to physical health (six items), physical endurance (six items), treatment satisfaction (four items), financial worries (four items) and emotional/mental health (five items). This tool has also been used by other studies, including in Uganda.^27^ A face validity test was done to ensure the appropriateness of the tool for measuring QoL among PWDM in the context of our study. The tool was pretested on 20 selected non-expert PWDM. A reliability test produced a Cronbach’s α of 0.76, which indicated internal consistency of domain items and therefore rendered the tool appropriate.^28^ A translated online version of the tool was also developed and pretested through back-translation to ensure consistency with the translated version.^29^

### Administering the diabetes-related QoL questionnaire

The questionnaire was scored against various aspects of a person’s perceived QoL before the COVID-19 pandemic, during the COVID-19 pandemic and after the end of the pandemic. Although the tool is usually self-administered, we used an interviewer-administered approach due to the difficulty in reading associated with older age groups, visual problems common with diabetes patients and the low literacy levels among the adult rural population in Uganda. To limit response order and acquiescence biases that tend to be associated with Likert responses and self-administered tools,^30^ the interviewer (WS) allowed a 10-min session break between diabetes-related QoL assessments across the three time periods and, where appropriate, altered the sequence of administering the QoL survey questions.

### Description of variables

#### Primary outcome variable

Diabetes-related QoL was the primary outcome. This was self-reported and scored against a 25-item, 125-point questionnaire categorised under five domains. The domains were role limitation (six items), physical endurance (six items), treatment satisfaction (four items), financial worries (four items) and emotional/mental health (five items). Each item was measured on a 5-point Likert scale.

#### Secondary outcome variables

For each participant, we captured sociodemographic as well as lifestyle and clinical characteristics. Sociodemographic characteristics were age (years), sex (male/female), marital status, education level, occupation and source of income. Lifestyle and clinical characteristics were duration of diabetes, diabetes treatment, source of diabetes care, distance from care centre, clinic visit frequency, mode of transport, presence of diabetes complications, existing comorbidity, coronavirus vaccination and infection, tobacco and alcohol use and diabetes self-management practices.

### Data analysis

We used SPSS Windows version 28.0 (IBM, Armonk, NY, USA) statistical software for analysis. A p-value <0.05 was used as the standard cut-off for statistical significance in all correlation and regression analyses.

#### Scoring of diabetes-related QoL

Domain-specific scores were computed as summated points earned from individual domain items. Diabetes-related QoL for each participant was then computed as the fraction of the total sum of all domain-specific scores and the expected maximum score (125). The fractions were multiplied by 100 to standardise reporting and comparisons of QoL scores. The internal validity of the diabetes-related QoL tool was assessed using Cronbach’s α, which yielded a value of 0.90. Domain-specific α values were role limitation (α=0.859), physical endurance (α=0.853), treatment satisfaction (α=0.778), financial worries (α=0.844) and emotion/mental health (α=0.645). The overall diabetes-related QoL score for each time period was presented as median (IQR) and categorised as satisfactory (≥60) and unsatisfactory (<60). A cut-off of <60 has been found to provide excellent sensitivity and negative prediction of QoL among older adults elsewhere.^31^ A test for normality of the overall QoL score was performed using the Shapiro–Wilk test, yielding a p-value <0.05 (rejected normal distribution).

#### Descriptive statistics

Proportions (%), number (n), median (interquartile range [IQR]) and mean (±standard deviation [SD]) were used to summarise descriptive statistics, where appropriate. Variations in descriptive statistics across sex categories were analysed using the χ^2^ test and Mann–Whitney U test for categorical and continuous variables as appropriate.

#### Difference in diabetes-related QoL across the time periods

We analysed the difference between the median overall diabetes-related QoL scores across the three time periods using Friedman’s analysis of variance. A post hoc Wilcoxon signed-rank test was performed to identify which of the three time periods were different. To avoid the possibility of declaring a false significant result of the Wilcoxon signed-rank test (type I error), a Bonferroni correction was applied. The Bonferroni correction yielded a p-value <0.017 (0.05/3), which was considered to reject the null hypothesis.

#### Factors associated with diabetes-related QoL

We analysed the sociodemographic, lifestyle and clinical factors associated with diabetes-related QoL using post-pandemic scores as the dependent variable. We performed logistic regression, where we included variables that yielded a p-value <0.25 at bivariate analysis and a priori variables documented in predictor models elsewhere.^32^ Odds ratios (ORs) and adjusted ORs (aORs) with 95% confidence intervals (CIs) were obtained. Key assumptions for our logistic model included independence of errors, absence of multicollinearity, lack of strongly influential outliers and missing data being ‘missing completely at random’. A p-value <0.05 was used to determine statistical significance.

## Results

### Participant characteristics

#### Sociodemographic characteristics

We analysed data from 410 participants, the majority of whom were females (68.3%) and were residing in the Sheema district (38.0%). The participants’ mean age was 58 y (SD 9.2), with no significant age difference between males and females (p=0.652). More males than females were married, had attained higher education, were employed and had means of mobility in their household (all p<0.001). Table 1 presents the sociodemographic characteristics of the participants.

**Table 1.** Sociodemographic characteristics of participants.

Characteristics	Overall	Male	Female	p-Value
District, n (%)				NS^a^
Sheema	156 (38.0)	43 (33.1)	113 (40.4)	
Isingiro	147 (35.9)	47 (36.1)	100 (35.7)	
Ntungamo	107 (26.1)	40 (30.8)	67 (23.9)	
Age (years), mean (SD)	58.0 (9.2)	58.9 (9.6)	57.6 (9.0)	NS^b^
Age group, n (%)				NS^a^
<50	76 (18.6)	21 (16.1)	55 (19.7)	
50–59	137 (33.4)	41 (31.5)	96 (34.2)	
60–69	149 (36.3)	50 (38.5)	99 (35.4)	
≥70	48 (11.7)	18 (13.9)	30 (10.7)	
Marital status, n (%)				<0.001^a^
Unmarried	133 (32.4)	14 (10.8)	119 (42.5)	
Married	277 (67.6)	116 (89.2)	161 (57.5)	
Education level completed, n (%)				<0.001^a^
None	235 (57.3)	60 (46.2)	175 (62.5)	
Primary	120 (29.3)	43 (33.1)	77 (27.5)	
Post-primary	55 (13.4)	27 (20.7)	28 (10)	
Employment, n (%)				<0.001^a^
Formal employment	22 (5.4)	16 (12.3)	06 (2.1)	
Informal casual worker	136 (33.1)	51 (39.2)	85 (30.4)	
Unemployed	252 (61.5)	63 (48.5)	189 (67.5)	
Possession of mobility means, n (%)				<0.001^a^
None	226 (55.1)	43 (33.1)	183 (65.4)	
One or more	184 (44.9)	87 (66.9)	97 (34.6)	

NS: non-significant difference.

a χ^2^ test.

b Mann–Whitney U test.

#### Clinical and lifestyle characteristics of participants

The participants had a mean duration of diabetes of 8.9 years (SD 6.3), with no significant difference between males and females. Most participants sought diabetes care on a monthly routine (93.9%) and lived at least 2 km from a government health facility with diabetes services (86.8%). Participants were mainly managed on monotherapy (93.9%) and the majority had a diabetes complication (69.2%) and a chronic disease condition other than diabetes (61.5%). Almost all participants had received a COVID-19 vaccination (92.2%), 90.7% of them having completed the two World Health Organization–recommended doses. The median diabetes-related QoL scores were comparatively lower during the pandemic, with no significant differences between males and females. Table 2 presents the clinical and lifestyle characteristics of the participants.

**Table 2.** Clinical and lifestyle characteristics of participants.

Characteristics	Overall	Male	Female	p-Value
Duration of diabetes (years), mean (SD)	8.9 (6.3)	9.2 (6.4)	8.8 (6.2)	NS^a^
Diabetes treatment, n (%)				NS^b^
One	385 (93.9)	119 (91.5)	266 (95.0)	
More than one	25 (6.1)	11 (8.5)	14 (5.0)	
Distance from the health centre, n (%)				NS^b^
<5 km	222 (54.1)	67 (51.5)	155 (55.4)	
≥5 km	188 (45.9)	63 (48.5)	125 (44.6)	
Mode of travel to the health centre, n (%)				0.004^b^
Non-motorized (foot/bicycle)	126 (30.7)	43 (33.1)	83 (29.6)	
Motorized (motorcycle/taxis)	284 (69.3)	87 (66.9)	197 (70.4)	
Presence of complications, n (%)				NS^b^
None	126 (30.8)	43 (33.1)	83 (29.7)	
One	142 (34.6)	45 (34.6)	97 (34.6)	
More than one	142 (34.6)	42 (32.3)	100 (35.7)	
Presence of chronic comorbidity, n (%)				0.009^a^
None	158 (38.5)	62 (47.7)	96 (34.3)	
One or more	252 (61.5)	68 (52.3)	184 (65.7)	
Contracted COVID-19				NS^b^
No	376 (91.7)	118 (90.8)	258 (92.1)	
Yes	34 (8.3)	12 (9.2)	22 (7.9)	
Obtained COVID-19 vaccine, n (%)				NS^b^
Yes	378 (92.2)	119 (91.5)	259 (92.5)	
No	32 (7.8)	11 (8.5)	21 (7.5)	
Completed COVID-19 vaccination (n=378), n (%)				NS^b^
Yes	343 (90.7)	105 (88.2)	238 (91.9)	
No	35 (9.3)	14 (11.8)	21 (8.1)	
Tobacco use (n=405), n (%)				NS^b^
Yes	17 (4.2)	07 (5.4)	10 (3.6)	
No	388 (95.8)	123 (94.6)	265 (96.4)	
Alcohol use, n (%)				<0.001^b^
Yes	47 (11.7)	29 (23.0)	18 (6.5)	
No	356 (88.3)	97 (77.0)	259 (93.5)	
QoL score, median (IQR)				
Pre-pandemic	67.2 (60.0, 75.2)	67.6 (60.6, 76.8)	66.8 (60.0, 74.4)	NS^a^
Pandemic	62.4 (54.4, 72.0)	62.4 (55.8, 72.4)	62.4 (54.4, 71.8)	NS^a^
Post-pandemic	68.8 (58.4, 76.0)	70.4 (60.6, 77.0)	68.0 (58.4, 75.2)	NS^a^

NS: non-significant difference.

a Mann–Whitney U test.

b χ^2^ test.

### Change in self-management practices before and during the pandemic

**Figure 1. (A):**
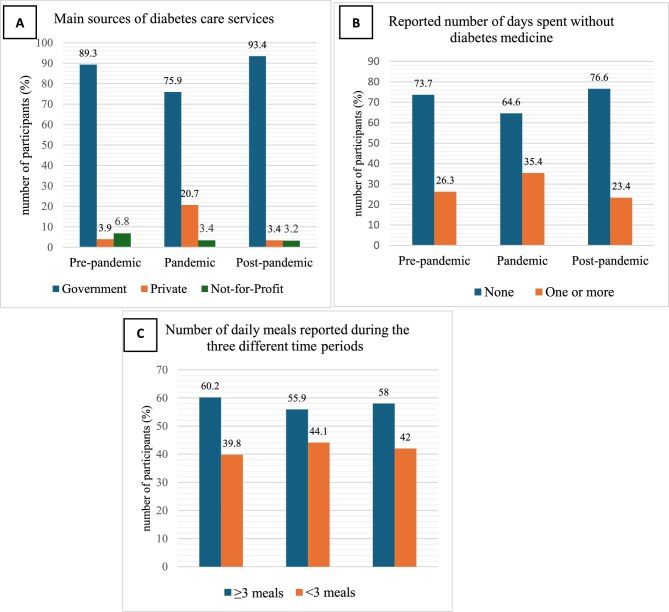
The changes in the type of health facility from which diabetes care was sought, **(B)** the days spent without individual prescription supplies and **(C)** daily meal frequency across the time periods.

The number of participants using private clinics increased about fivefold, from 16 (3.9%) pre-pandemic to 85 (20.7%) during the pandemic. Those who lived ≥1 d without medication increased from 108 (26.3%) before the pandemic to 145 (35.4%) during the pandemic. There was a slight increase in the number of participants who reported eating less than three meals a day, from 163 (39.8%) before the pandemic to 181 (44.1%) during the pandemic. The median frequency of days engaging in physical work or exercise was the same before and during the pandemic (5.0 [IQR 2–6]). No change in weekly self-monitoring of blood glucose before and during the pandemic was seen, with an equal proportion of participants (87.1%) reporting not having been doing weekly self-monitoring before the pandemic as during the pandemic. Figure 1 illustrates the changes in the type of health facility from which diabetes care was sought, the availability of prescription supplies and daily meal frequency across the periods.

### Self-reported diabetes-related QoL before, during and after the pandemic

The overall pre-pandemic, pandemic and post-pandemic median diabetes-related QoL scores were 67.2 (IQR 60.0–75.2), 62.4 (IQR 54.4–72.0) and 68.8 (IQR 58.4–76.0), respectively. There was a statistically significant difference in diabetes-related QoL (p<0.001) across the three time periods. The post hoc Wilcoxon signed-rank tests with Bonferroni correction (significant at p<0.017) indicated statistically significant differences in overall median diabetes-related QoL scores between the pre-pandemic and pandemic periods (Z=−13.08, p<0.001) and the pandemic and post-pandemic periods (Z=−8.55, p<0.001), but not between the pre-pandemic and post-pandemic periods (Z=−2.04, p=0.042). The QoL median scores during the pandemic were slightly but significantly lower than the pre-pandemic by 4.8 points and the post-pandemic by 6.4 points, which shows a mild difference in scores. The highest-scoring domain was physical endurance, whereas the lowest-scoring domain was financial worries. Table 3 presents the overall median diabetes-related QoL scores across the three time periods.

**Table 3.** Median QoL scores with proportions of satisfactory and unsatisfactory QoL before, during and after the pandemic

Variables	Pre-pandemic	Pandemic	Post-pandemic	p-Value
**Overall (n=410)**				
Overall D-QoL				
Median score (IQR)	67.2 (60.0–75.2)	62.4 (54.4–72.0)	68.8 (58.4–76.0)	<0.001^a^
Satisfactory (≥60), n (%)	320 (78.0)	252 (61.5)	303 (73.9)	<0.001^b^
Unsatisfactory (<60), n (%)	90 (22.0)	158 (38.5)	107 (26.1)	0.047^c^
**Domain-specific D-QoL (n=410)**				
Role limitation				
Median score (IQR)	66.7 (56.7–80.0)	61.7 (50.0–80.0)	66.7 (53.3, 80.0)	<0.001^a^
Satisfactory (≥60), n (%)	291 (71.0)	238 (58.0)	275 (67.1)	<0.001^b^
Unsatisfactory (<60), n (%)	119 (29.0)	172 (42.0)	135 (32.9)	NS^c^
Physical endurance				
Median score (IQR)	76.7 (66.7–86.7)	76.7 (60.0–86.7)	73.3 (60.0–86.7)	<0.001^a^
Satisfactory (≥60), n (%)	351 (85.6)	322 (78.5)	319 (77.8)	<0.001^b^
Unsatisfactory (<60), n (%)	59 (14.4)	88 (21.5)	91 (22.2)	<0.001^c^
Treatment satisfaction				
Median score (IQR)	70.0 (60.0–80.0)	67.5 (55.0–80.0)	75.0 (65.0–80.0)	<0.001^a^
Satisfactory (≥60), n (%)	317 (77.3)	271 (66.1)	349 (85.1)	<0.001^b^
Unsatisfactory (<60), n (%)	93 (22.7)	139 (33.9)	61 (14.9)	<0.001^c^
Financial worries				
Median score (IQR)	45.0 (40.0–60.0)	40.0 (35.0–50.0)	50.0 (40.0–65.0)	<0.001^a^
Satisfactory (≥60), n (%)	123 (30.0)	68 (16.6)	166 (40.5)	<0.001^b^
Unsatisfactory (<60), n (%)	287 (70.0)	342 (83.4)	244 (59.5)	<0.001^c^
Emotional/mental stress				
Median score (IQR)	68.0 (60.0–76.0)	64.0 (56.0–76.0)	72.0 (60.0–80.0)	<0.001^a^
Satisfactory (≥60), n (%)	316 (77.1)	280 (68.3)	328 (80.0)	<0.001^b^
Unsatisfactory (<60), n (%)	94 (22.9)	130 (31.7)	82 (20.0)	NS^c^

D-QoL: diabetes-related QoL; NS: non-significant.

a Friedman test.

b McNemar pre-pandemic vs pandemic test.

c McNemar pre-pandemic vs post-pandemic test.

Compared with the pre-pandemic period (22.0%), the overall proportion of participants with unsatisfactory diabetes-related QoL (median score <60) was significantly higher in the pandemic (38.5%, p<0.001) and post-pandemic (26.1%, p=0.047) periods. Across all domains there was an increased proportion of participants with unsatisfactory QoL during the pandemic than before (p<0.001). The McNemar test further showed that all but the emotional/mental stress and role limitation domains had significant differences between the pre-pandemic and post-pandemic periods. Table 3 presents the proportion of participants with unsatisfactory and satisfactory diabetes-related QoL.

### Factors associated with diabetes-related QoL among participants

As shown in Table 4, participant age, diabetes complications, presence of comorbidity, education level and travel mode for care were all significantly associated with QoL at bivariate logistic regression (all p<0.05). At multivariate logistic regression, only diabetes complications, presence of comorbidity, travel mode for care and education were significantly associated with QoL (all p<0.05). The odds of having an unsatisfactory QoL in participants with diabetes complications (aOR 4.3 [95% CI 2.1 to 8.8], p<0.001) and chronic comorbidity (aOR 2.1 [95% CI 1.2 to 3.8], p=0.012) were higher compared with those without. Participants with no formal education (aOR 2.4 [95% CI 1.4 to 4.4], p<0.003) and who travelled for care using non-motorised means (aOR 3.9 [95% CI 2.3 to 6.7], p<0.001) had higher odds of reporting unsatisfactory QoL compared with those with formal education and who used motorised means, respectively.

**Table 4.** Factors associated with unsatisfactory post-pandemic QoL among participants

Category	OR (95% CI)	p-Value	aOR^a^ (95% CI)	p-Value
Age group (years)				
>50	1.0	Ref	1.0	Ref
50–59	1.1 (0.5 to 2.2)	NS	0.3 (0.1 to 1.1)	NS
60–69	1.9 (0.9 to 3.7)	NS	0.2 (0.0 to 1.2)	NS
≥70	3.7 (1.7 to 8.4)	0.001	0.2 (0.0 to 2.4)	NS
Sex				
Male	1.0	Ref	1.0	Ref
Female	1.2 (0.7 to 1.9)	NS	1.0 (0.6 to 1.9)	NS
DM complications				
None	1.0	Ref	1.0	Ref
One or more	4.8 (2.5 to 9.1)	<0.001	4.3 (2.1 to 8.8)	<0.001
Presence of comorbidity				
None	1.0	Ref	1.0	Ref
One or more	2.7 (1.7 to 4.6)	<0.001	2.1 (1.2 to 3.8)	0.012
Employment				
Employed	1.0	Ref	1.0	Ref
Unemployed	1.6 (1.0 to 2.5)	NS	1.1 (0.6 to 1.9)	NS
Ownership of mobility means				
One or more	1.0	Ref	1.0	Ref
None	1.5 (1.0 to 2.4)	NS	1.0 (0.5 to 1.6)	NS
Primary mode of travel for care				
Motorised (motorcycles/taxis)	1.0	Ref	1.0	Ref
Non-motorized (foot/bicycle)	3.5 (2.2 to 5.5)	<0.001	3.9 (2.3 to 6.7)	<0.001
Education level				
Educated	1.0	Ref	1.0	Ref
None	2.8 (1.7 to 4.6)	<0.001	2.4 (1.4 to 4.4)	0.003

Ref: reference group; NS: non-significant difference.

a Adjusted for age, sex, diabetes duration, diabetes complications, comorbidity, employment, ownership of mobility means, mode of travel for care and education.

## Discussion

Our study aimed to report the impact of the COVID-19 pandemic on the QoL of adult PWDM in rural Uganda using a quantitative patient survey. To our knowledge, this is the first study in Uganda to report the QoL in rural diabetes patients since the global declaration of the COVID-19 pandemic.

### Main findings

Our findings reveal that the COVID-19 pandemic was associated with a significant deterioration in the QoL among rural PWDM in Uganda, evidenced by significantly lower diabetes-related QoL scores during the pandemic than in any other period. There was a 75% significant increase in PWDM with unsatisfactory diabetes-related QoL during the pandemic. Having diabetes complications, chronic comorbidity, no formal education and seeking healthcare using non-motorised transport (foot or cycling) were all significantly independently associated with unsatisfactory diabetes-related QoL. We further observed that the diabetes patient utilisation of private clinics increased fourfold during the pandemic compared with the pre-pandemic figure. The number of PWDM who spent at least 1 d without diabetes prescription supplies increased by 35% during the pandemic compared with before.

### Variations of QoL among PWDM in rural Uganda during the three periods

The QoL of rural PWDM significantly declined during the pandemic, with more than a third of participating PWDM saying it was unsatisfactory. This can be attributed to the COVID-19-related measures imposed that resulted in care accessibility barriers, social isolation, loss of income and food scarcity.^33^ This may have affected the PWDM’s treatment continuity and affordability of other life needs, resulting in poor adherence to diabetes management recommendations and treatment. First, rural settings in Uganda inherently suffer inequality in the distribution, quality and availability of diabetes care services, which is believed to have worsened during the pandemic due to the prioritisation of COVID-19 over other disease care.^11,19,34^ Second, rural settings also have disproportionately high poverty rates, which, given the economic disruption occasioned by the pandemic, may have worsened the financial capacity of PWDM to afford alternatives.^10^ Such uncertainty may have affected their ability to fulfil their disease needs, resulting in unsatisfactory QoL.

Interestingly, the fact that there were significant differences in pre- and post-pandemic overall QoL median scores is an indication that this rural population of PWDM was able to generally overcome the pandemic’s negative impacts to regain their initial state of perceived QoL. This was also realised with the mental and role aspects of QoL, demonstrating that PWDM regained their emotional and mental status quo, as well as their ability to perform their various roles as before the pandemic. However, the same cannot be said for the specific QoL aspects of physical endurance, treatment satisfaction and financial worries. This may have been due to the deterioration in disease processes over time that weakened individual physical endurance, and a possible delay in the healthcare system and economic recovery after the pandemic, which prolonged treatment and financial challenges.^35,36^

### Factors associated with post-pandemic QoL among PWDM in rural Uganda

In this rural population of PWDM, having diabetes complications and chronic comorbidity were independently negatively associated with QoL. PWDM with complications and chronic comorbidity are four times and two times, respectively, more likely to be unsatisfied with their QoL than those without. Similar findings have been reported in quantitative studies using generalised regression models in Ethiopia^37,38^ and India.^14^ The debilitating nature of diabetes-related complications or the presence of chronic comorbidity constrains an individual’s performance of daily routines, causes physical pain, increases treatment burden and worsens helplessness, leading to a poorly perceived QoL.

We also observed significantly higher odds of being unsatisfied with the QoL among patients using non-motorised transport to seek care and those with no formal education. This can be explained by the relatively higher socio-economic status associated with being educated and affording to own the means or the cost of paying for motorised means. Nevertheless, we observed a positive response to COVID-19 vaccination in this rural diabetes population, which portrays their positive response to healthcare programs and is an indication of the potential for the success of diabetes-related community programs.

### Variations in diabetes self-management practices in rural Uganda over the three periods

We observed an increased use of private clinics during the pandemic, which points to a possible coping strategy that may have arisen from inaccessibility or a lack of diabetes treatment supplies in government health facilities. With the government health facilities usually located far away, it would have been costly for patients to access, given the high travel costs that characterised the pandemic.^39^ This, given the unguaranteed availability of drugs in government facilities, may have prompted PWDM to prefer nearby private clinics, using the money they would have paid for transport to government health centres. Government health facilities were affected by severe disruptions in non-COVID-19 medical supplies, which created severe shortages of diabetes treatment supplies.^18^

Owing to financial constraints that characterised the pandemic and as evidenced by very low financial domain scores, many PWDM may not have been able to keep up with the cost of diabetes prescriptions. This explains the observed increase in patients going days without medication during the pandemic compared with before. With the severe lack of diabetes supplies in government health facilities that characterised the pandemic disruptions and the unaffordable prices at private care alternatives, treatment adherence could not be guaranteed. Poor treatment adherence is a major cause of poor diabetes control and a risk factor for diabetes-related complications, morbidity and premature mortality.^40^ Therefore, it is plausible to argue that diabetes control may have generally deteriorated during the pandemic in rural Uganda, contributing to the deterioration in QoL. This is supported by studies from sub-Saharan Africa that have reported the pandemic worsened diabetes control.^41,42^

### Strengths and limitations

Our study presents the first health-related QoL analysis among rural diabetes patients. We provide QoL scores over a broad time period, representing the pre-pandemic, pandemic and post-pandemic periods. This provided a picture of the variations in QoL to depict the impact of the pandemic. However, there were some limitations to the study. First, we assessed the QoL over a more extended period across three time periods. This self-reported account required participants to recall experiences and perceptions stretching across these different periods, which may have been associated with some recall bias. Thus, the pre-pandemic and pandemic measures may not represent the actual perception if they were interviewed in real time. The authors attempted to minimise this bias that may have been associated with administering the tool, as reported in the Methods. However, this may not guarantee absolute mitigation of the associated recall or social desirability bias in the responses.

We provide an analysis of factors associated with diabetes-related QoL using the post-pandemic scores as the dependent variable. This does not reflect the factors associated with diabetes-related QoL before and during the pandemic. However, this would have provided a better picture of how the factors evolved over the time periods. Nevertheless, we recruited a homogeneous sample from three districts, which strengthens the generalisability of our findings to rural country regions and low-income country settings with similar healthcare system functioning, socio-economic characteristics and COVID-19 responses.

## Conclusions

The COVID-19 pandemic worsened the QoL of people with diabetes in rural Uganda, with considerable deterioration in financial worries, role limitations due to diabetes and treatment satisfaction. The disruptions in social, economic and healthcare access occasioned by the COVID-19 prevention measures affected the well-being and choices of people with diabetes, impacting their social, emotional, financial and physical well-being, which defined their perception of daily treatment and disease management processes. The socio-economic and healthcare needs of people with diabetes should be prioritised in the development and implementation of epidemic policies. The government should increase diabetes care resources at public healthcare facilities and also partner with private healthcare providers in enhancing the accessibility of diabetes care services during epidemics and pandemics.

## Data Availability

The data underlying this article will be shared upon reasonable request to the corresponding author.
